# Dysuria, heat stress, and muscle injury among Nicaraguan sugarcane workers at risk for Mesoamerican nephropathy

**DOI:** 10.5271/sjweh.3963

**Published:** 2021-06-29

**Authors:** Tiffany L Stallings, Alejandro Riefkohl Lisci, Nathan L McCray, Daniel E Weiner, James S Kaufman, Ann Aschengrau, Yan Ma, Michael P LaValley, Oriana Ramírez-Rubio, Juan Jose Amador, Damaris López-Pilarte, Rebecca L Laws, Michael Winter, V Eloesa McSorley, Daniel R Brooks, Katie M Applebaum

**Affiliations:** Department of Environmental and Occupational Health, Milken Institute School of Public Health, The George Washington University, Washington, DC, USA; Department of Epidemiology, Boston University School of Public Health, Boston, MA, USA; Division of Nephrology, Tufts Medical Center, Boston, MA, USA; Division of Nephrology, VA New York Harbor Healthcare System and New York University School of Medicine, New York, NY, USA; Department of Biostatistics and Bioinformatics, Milken Institution School of Public Health, The George Washington University, Washington, DC, USA; Department of Biostatistics, Boston University School of Public Health, Boston, MA, USA; Barcelona Institute for Global Health, ISGlobal, Barcelona, Spain; Department of Environmental Health, Boston University School of Public Health, Boston, MA, USA; Biostatistics and Epidemiology Data Analytics Center, Boston University School of Public Health, Boston, MA, USA; Department of Public Health Sciences, University of Chicago, Chicago, IL, USA

**Keywords:** chronic kidney disease of unknown origin, CKDu, crystalluria, chronic kidney disease, rhabdomyolysis

## Abstract

**Objectives:**

Nicaraguan sugarcane workers, particularly cane cutters, have an elevated prevalence of chronic kidney disease of unknown origin, also referred to as Mesoamerican nephropathy (MeN). The pathogenesis of MeN may include recurrent heat stress, crystalluria, and muscle injury with subsequent kidney injury. Yet, studies examining the frequency of such events in long-term, longitudinal studies are limited.

**Methods:**

Using employment and medical data for male workers at a Nicaraguan sugarcane company, we classified months of active work as either work as a cane cutter or other sugarcane job and determined occurrence of dysuria, heat events and muscle events. Work months and events occurred January 1997 to June 2010. Associations between cane cutting and each outcome were analyzed using logistic regression based on generalized estimating equations for repeated events, controlling for age.

**Results:**

Among 242 workers with 7257 active work months, 19.5% of person-months were as a cane cutter. There were 160, 21, and 16 episodes of dysuria, heat events, and muscle events, respectively. Compared with work months in other jobs, cane cutting was associated with an elevated odds of dysuria [odds ratio 2.40 (95% confidence interval 1.56–3.68)]. The number of heat and muscle events by cane cutter and other job were limited.

**Conclusions:**

Working as a cane cutter compared with other jobs in the sugarcane industry was associated with increased dysuria, supporting the hypothesis that cane cutters are at increased risk of events suspected of inducing or presaging clinically evident kidney injury.

Chronic kidney disease (CKD) not explained by typical CKD risk factors is exceedingly common along the Pacific coast of countries in Central America, including Nicaragua, El Salvador, Guatemala, and Costa Rica (1). This region includes the location of our present study in northwestern Nicaragua, where CKD prevalence is particularly high among young (age 30–40 years) male agricultural laborers, principally sugarcane industry workers (2–8). This distinct regional pattern of CKD is referred to as Mesoamerican nephropathy (MeN).

Among sugarcane workers, cane cutters appear at greatest risk of MeN (6, 9–12). One of the main proposed causal hypotheses has focused on the potential physical challenges faced by cane cutters, including strenuous physical exertion in extreme heat (3, 13–18), such that volume depletion in conjunction with other factors, such as muscle injury, results in recurrent episodes of subclinical kidney injury that eventually manifest with clinically apparent CKD (13–19).

Dysuria, defined as pain with urination and called “chistata” in Nicaragua, appears common among male sugarcane workers, and affected male workers may be misdiagnosed as having urinary tract infections (UTI) (20, 21). Urinalyses of cane cutters demonstrate crystalluria (10, 14, 20, 22), with increasing crystal burden over a work shift (22). One hypothesis is that dysuria reflects mechanical trauma from crystalluria, influenced in part by work-related heat stress and volume depletion with resultant highly concentrated urine (14, 15, 20, 22, 23).

With demanding physical exertion, cane cutters may also be at risk of muscle damage with release of nephrotoxic proteins from muscle, including myoglobin (13, 14, 17–19, 24–26). Elevated concentrations of muscle injury biomarkers, myoglobin and creatinine kinase (CK), are associated with muscle damage-related illness and acute kidney injury (AKI) (24–27). Two studies reported that cane cutters experienced an increase in serum CK across a workday (19, 28), suggesting a possible role for muscle injury in MeN. Importantly, although cane cutters have been the focus of much of the MeN research, data are lacking on whether workers holding other jobs in the sugarcane industry may also experience events of dysuria, heat events, or muscle injury.

Prior research in populations at risk of MeN is limited by short-term study designs and reliance on self-report of work and medical events. In the current study, we combined employment history and medical records to create a 13-year longitudinal retrospective cohort to examine whether cane cutters compared with workers in other jobs in the sugarcane industry sought medical care for dysuria and conditions related to heat stress and muscle injury.

## Methods

### Study population

Data were collected from a sugarcane plantation in northwestern Nicaragua, Department of Chinandega for a feasibility study of sugarcane workers to evaluate the potential for conducting multi-year retrospective studies in the region using employment and medical records [detailed in (29)]. Briefly, employment records were used to identify men who actively worked between 1 January 1997 and 30 June 2010. For each calendar year, workers were randomly sampled within specific job types (cane cutter, irrigator, pesticide applicator, field machine operator, factory worker, and cane gatherer), with larger sampling of cane cutters (sampled 7 cane cutters per year and 2–3 workers in the other job categories per year, approximately), yielding a total of 243 workers. Workers’ complete medical records were then abstracted and medical events through 30 June 2010 were determined. For the present analysis, we excluded one worker whose data indicated he did not work during the study period, leaving 242 male workers. Below, we describe our analysis by person-month, examining whether workers received medical care for dysuria, heat events, or muscle events, and whether these were more likely to occur while working as a cane cutter or other job in the sugarcane industry.

### Work classification

For these workers, employment and payroll records provided work history, including date of initial hire and dates held different jobs. For each calendar month from each workers’ date sampled for the study until the end of follow-up 30 June 2010, we determined whether the worker was actively working. Only active work months were used in this analysis and each person-month was classified as time worked as a cane cutter or other job in the sugarcane industry, providing time-varying classification of job type.

### Medical records

Prior data collection involved locating medical records from the onsite medical clinic, where workers receive their healthcare while working on the plantation. Data abstracted from medical records included symptoms, physician diagnoses, prescriptions, vital signs, and blood and urine laboratory results, including serum creatinine. In the medical records, information on lifestyle behaviors such as alcohol consumption and smoking were limited to 33 workers (17.4%). Medical records were abstracted in Spanish. For the present work, two native Spanish-speaking physicians translated the data to English – one a member of our team (ARL), the other at a translation service company.

Because this analysis was not focused on development of CKD but rather incidence of potential CKD precursor events by job type, we excluded person-time after a worker developed CKD (N=8 workers). For the eight workers, a total of 55 work months starting with the month of CKD diagnosis and later were excluded from the study follow-up period. We considered a worker to have CKD during the observation period if there was a physician diagnosis, which was confirmed with two creatinine-based estimated glomerular filtration rate (eGFR) values <60 mL/min per 1.73 m^2^ at least three months apart estimated using the CKD-EPI equation for non-black men (30, 31). Hypertension was defined by physician diagnosis accompanied by a prescription for antihypertensive medication or elevated systolic or diastolic blood pressure (≥140 and/or ≥90 mmHg, respectively) and diabetes by physician diagnosis accompanied by prescription for diabetic medication or serum glucose >200 mg/dl (assumed to be non-fasting).

The Institutional Review Boards at George Washington University and Boston University Medical Center and the Nicaraguan Ministry of Health [Ministerio de Salud de Nicaragua (MINSA)] approved this study.

### Classification of medical outcomes

Our classification of the three study outcomes (dysuria, heat events, and muscle events) entailed compiling symptoms and/or diagnoses that described the potential occurrence of each event. For dysuria, we identified events of pain upon urination. If a medical visit mentioned pain with urination, dysuria, chistata, cystitis, or UTI (a common misdiagnosis with later evidence supporting these were likely crystalluria, discussed in the introduction), then the visit was reviewed as a potential dysuria event. For both heat and muscle events, we describe events meeting primary and secondary definitions of these outcomes. The primary definition of heat events focused on medical visits in which a worker was noted to have overheated. If a medical visit specifically mentioned heat stress, heat stroke, overheated, hyperthermia, dehydration, or dizziness, then the visit was reviewed as a potential primary heat event. Also, we reviewed the visit as a potential primary heat event if it indicated physical exhaustion or fatigue combined with at least one heat symptom (headache, muscle cramp or spasm, nausea, vomiting, asthenia/weakness, tiredness). For the secondary definition of a heat event, we considered as a potential event those visits with terms indicating physical exhaustion or fatigue (without accompanying heat symptoms), electrolyte imbalance, or experiencing ≥2 heat symptoms. We note that medical visits were reviewed in full if any one of the heat symptoms was indicated, though one heat symptom alone was considered insufficient to meet even the secondary definition. The primary definition of muscle event focused on identifying symptoms possibly related to rhabdomyolysis, a type of serious muscle injury often due to high physical exertion. Visits indicating myalgia or diffuse, non-specific muscle injury/pain or discomfort were reviewed as a potential event. The secondary definition described body pain or difficulty moving, or reddish/tea-colored urine, a potential indicator of myoglobinuria due to rhabdomyolysis.

**Figure 1:**
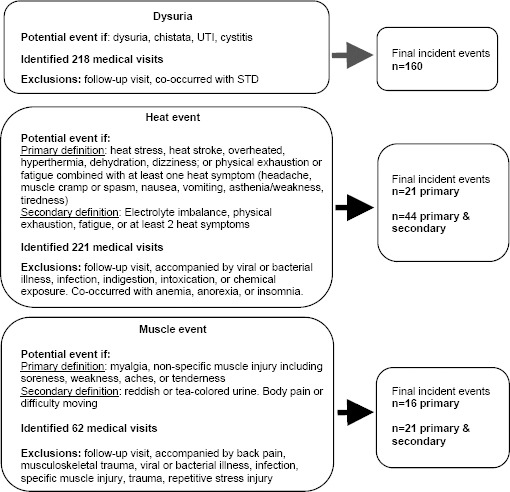
Process for classifying occurrence of three medical outcomes of interest in male Nicaraguan sugarcane workers: dysuria, heat events, and muscle events. The medical records may not state these events specifically; therefore, we searched for medical visits that indicated symptoms and diagnoses consistent with the events of interest. For heat and muscle events, we created primary and secondary definitions. For all three events, another incident event of the same type could not occur until a month passed. [UTI=urinary tract infection; CKD=chronic kidney disease; STD=sexually transmitted disease]

We then applied exclusion criteria. The main reasons for excluding events were follow-up visits to check the status of the worker’s health after the initial visit or co-occurred with an illness during the same visit (eg, for dysuria specifically, excluded if a sexually transmitted disease was indicated at visit; for heat or muscle event, excluded if an accompanying viral or gastrointestinal illness). A worker could experience each outcome more than once; however, only one event of each type could be counted within a calendar month. Also, another incident event of the same type could only be included after a 28-day window had passed (eg, if an incident dysuria occurred on 31 October that meant that another incident dysuria event could only be included if it occurred after 28 November). Follow-up visits for a prior event (eg, rather than a new event) were also excluded. Information on the identification of events and exclusion criteria are shown in Figure 1. Two study team members conducted an independent review of the records and were blinded to employment history. They met to resolve discrepancies.

There were 1174 medical visits during working months in total during the observation period, representing 924 person-months. All visits were reviewed for potential indicators of each of the events of interest. Of these visits, 416 (occurring in 359 person-months) had terms or phrases related to one or more of the events and these visits were examined further to determine whether they met the definition for the event(s) and if they had any exclusion criteria (the remaining 758 medical visits had no terms or phrases related to the events). Through the review of medical records, 218 (181 person-months), 221 (209 person-months), and 62 (60 person-months) medical visits were determined to have conditions identifying potential dysuria events, heat events, and muscle events, respectively. Note that there were 334 visits evaluated for only one potential type of event and 82 visits evaluated for more than one potential event type. After applying exclusion criteria, there were determined to be 160 dysuria, 44 heat events (21 primary definition), and 22 muscle events (16 primary definition), which occurred during 209 work months. Our analysis structure was based on calendar month and with only one event of each type allowed to occur during a calendar month, the number of events dropped for muscle events only, leaving 160 dysuria, 44 heat events, and 21 muscle events occurring during 209 person-months. We also note that in our examination of heat event specifically, we identified a large number of potential events, yet many of these had only one potential symptom of heat event which was determined to be insufficient to meet the definition of a heat event in our analysis and were excluded.

### Statistical analyses

Demographic, occupational, and medical characteristics were described using frequencies for categorical variables and medians [interquartile range (IQR)] for continuous variables. We multiply imputed baseline age (N=10 imputations) using PROC MI in SAS when missing (N=27 workers), conditional on years since hired, cumulative months worked at baseline, and whether working as a cane cutter at baseline. Characteristics of ever and never cane cutters during study follow-up were compared using Chi-square tests/Fisher’s exact tests (if an expected cell count <5), and Wilcoxon rank sum tests for categorical and continuous variables, respectively.

As described earlier, workers who worked between 1 January 1997 and 30 June 2010 were randomly selected within job and year and only calendar months actively worked from the date sampled until they were no longer employed or until 30 June 2010 were included in the analysis. Therefore, for the follow-up period of January 1997 through June 2010, start of follow-up for each worker began with the first month of work on or after sampling date. Each at-risk person-month was classified by our exposure of interest, working as a cane cutter or working in another job in the sugarcane industry; individual workers could contribute person-time to both categories depending on their work history. Dysuria, heat events, and muscle events were treated as individual outcomes.

We compared the frequency of dysuria, heat events, or muscle events by months worked as a cane cutter or other job in the sugarcane industry, using Chi-square tests/Fisher’s Exact tests. We calculated the proportion of person-months in which one of the event types occurred for cane cutters and other jobs. To examine potential associations between working as a cane cutter and odds of each binary outcome, we performed a set of logistic regression analyses based on the generalized estimating equations (GEE) method (32) to account for correlation of repeated occurrences. We estimated odds ratios (OR) and 95% confidence intervals (CI). Younger workers were more likely to work as a cane cutter than other job and more likely to experience dysuria, heat, and muscle events. We generated models with and without controlling for age (continuous). In models controlling for age, we used PROC MIANALYZE to account for imputed age. Hypertension and diabetes were considered potential confounders, but few workers were diagnosed with these conditions and adjustment did not change the associations, therefore they were not included in final models.

The analytical dataset (N=242) contained 199 workers who had available medical records, of which 160 workers had a medical visit during follow-up (eg, working month between date of sampling through June 30, 2010). There were 43 workers who had no medical record over their entire time of employment. This was assumed to be due to those workers not seeking medical attention for any reason (including not experiencing any of the events of interest) and therefore included in the analysis. We compared characteristics of workers who did and did not have medical records to identify any differences. We repeated the analyses of the events of interest and working as a cane cutter, restricting to subjects who had a medical record to see if this influenced the results.

We also examined whether workers held the job of seed cutter at any point during the work months included in this analysis. Seed cutter is a job that involves climatic heat exposure and heavy physical exertion, similar to cane cutter, conditions that may place workers at increased risk of CKD or potential CKD precursors (11). Although the initial data collection did not sample for the job of seed cutter, a review of work history data identified that a limited number of workers held this job during study follow-up (eg, due to job changes). Therefore, we examined the proportion of work months in which dysuria was experienced among seed cutters to see how that may have influenced our results. All analyses were generated in SAS version 9.4 (SAS Institute, Cary, NC).

## Results

Over 13.5 calendar years, workers accrued 1417 months as cane cutters and 5840 months in other jobs. During follow-up, workers worked a median of 23 months and typically held one job (table 1). There were 93 workers who ever worked as a cane cutter during the follow-up period and their median time worked was lower than those who never worked as a cane cutter during follow-up (16 and 29 months, respectively).

**Table 1:** Characteristics of male Nicaraguan sugarcane workers. [IQR=interquartile range.]

Characteristic			Cane cutter ^a^				P-value ^b^
		
	Overall N=242		Ever N=93		Never N=149		
		
	N (%)	Median (IQR)	N (%)	Median (IQR)	N (%)	Median (IQR)	
Characteristics at baseline ^c^							
Birthplace							
Chinandega Department	135 (81.82)		33 (80.49)		102 (82.26)		0.25
Leon Department	18 (10.91)		3 (7.32)		15 (12.10)		
Other	12 (7.27)		5 (12.20)		7 (5.65)		
Age, years							
<25	70 (32.56)		30 (41.10)		40 (28.17)		0.07
25–32	75 (34.88)		26 (35.62)		49 (34.51)		
≥33	70 (32.56)		17 (23.29)		53 (37.32)		
Employment characteristics during follow-up ^d^							
Work months		23 (31)		16 (26)		29 (31)	<0.001
Work months as a cane cutter		0 (6)		10 (15)		0 (0)	<0.001
Number of jobs held		1 (1)		1 (1)		1 (1)	0.21

a Ever a cane cutter at any time during the follow-up period.

b P-values comparing characteristics between ever and never cane cutters: Wilcoxon rank sum test for continuous and Chi-square test for categorical characteristics.

c Missing birthplace for 77 workers and missing age for 27 workers.

d Characteristics during follow-up are analyzed using one observation per worker (eg, cumulative number of work months a worker contributed during follow-up).

During follow-up, there were 924 active work months with medical visits, and 66% of workers had at least one medical visit (table 2). Never cane cutters were more likely than ever cane cutters to seek medical care (P<0.001). There were a total of 160 dysuria events, 21 heat events, and 16 muscle events based on primary definitions. One-third of workers sought care for dysuria at least once. Workers who ever cut cane during follow-up experienced a higher median number of dysuria events compared with the never cane cutters (P=0.04). Based on primary definitions, 7% of workers experienced heat events and almost 6% experienced muscle events. Inclusion of secondary definitions more than doubled the number of heat events and modestly increased the number of muscle events (table 2).

**Table 2:** Medical events during follow-up. [CKD=chronic kidney disease; IQR=interquartile range.]

Characteristic ^a^			Cane cutter ^b^				P-value ^c^
		
	Overall N=242		Ever N=93		Never N=149		
		
	N (%)	Median (IQR)	N (%)	Median (IQR)	N (%)	Median (IQR)	
Traditional CKD risk factors							
Hypertension	10 (4.13)		1 (1.08)		9 (6.04)		0.09
Diabetes	3 (1.24)		0 (0)		3 (2.01)		0.29
Medical visits ^d^							
Workers with >1 visit	160 (66.12)		47 (50.54)		113 (75.84)		<0.001
Visits per worker ^e^		4.5 (6)		4 (7)		5 (5)	0.78
Medical outcomes ^f^							
Dysuria (N=160 events)							
Workers ^g^	80 (33.06)		35 (37.63)		45 (30.20)		0.23
Events per worker		1 (2)		2 (3)		1 (1)	0.04
Heat events							
Primary definition (N=21 events)							
Workers ^g^	18 (7.44)		9 (9.68)		9 (6.04)		0.29
Events per worker		1 (0)		1 (0)		1 (0)	0.17
Primary & secondary definitions (N=44 events)							
Workers ^g^	32 (13.22)		13 (13.98)		19 (12.75)		0.78
Events per worker		1 (1)		1 (0)		1 (1)	0.53
Muscle events							
Primary definition (N=16 events)							
Workers ^g^	14 (5.79)		4 (4.30)		10 (6.71)		0.43
Events per worker		1 (0)		1 (0)		1 (0)	0.42
Primary & secondary definitions (N=21 events)							
Workers ^g^	18 (7.44)		5 (5.38)		13 (8.72)		0.33
Events per worker		1 (0)		1 (0)		1 (0)	0.88

a Characteristics that occurred during follow-up, though diagnosis of traditional CKD risk factors may have occurred prior to follow-up.

b Ever a cane cutter at any time during the follow-up period.

c P-values comparing characteristics between ever and never cane cutters: Wilcoxon rank sum test for continuous characteristics; Chi-square or Fisher’s exact test (for hypertension and diabetes) for categorical characteristics.

d Medical visit could be for any reason, including reasons other than dysuria, heat event, or muscle event.

e For 160 workers with any medical visit during follow-up, they had a combined 5,843 work months in which a total of 924 months involved a medical visit.

f Only one medical event of each type (e.g., dysuria) could be counted within a work month.

g Workers with at least one medical event of that type (eg, dysuria). Events per worker are among workers who experienced at least one event of that type.

The proportion of months in which dysuria and heat events (primary definition) occurred were higher for cane cutters compared with those working in other sugarcane jobs (table 3). No differences in proportion of months experienced muscle events were apparent between the job categories. In logistic regression models, the odds of dysuria among cane cutter months were elevated compared with months worked in other jobs [adjusted OR 2.40 (95% CI 1.56–3.68)]. There were low numbers for primary heat and muscle events when grouped by cane cutter or other job and confidence intervals around the measures of association were wide, though the association with heat events did suggest that cane cutters were at elevated odds of experiencing these events. We noted earlier that not all workers had medical records and they were assumed to not have sought medical attention and therefore did not experience the events of interest. We compared characteristics for workers who did (N=199) and did not (N=43) have a medical record (supplementary material, www.sjweh.fi/article/3963, table S1). Those with a medical record were older, worked more total work months during follow-up (25 versus 11 median months), and were less likely to have ever been a cane cutter during follow-up compared with workers who did not have a medical record. In sensitivity analyses restricted to the 199 workers who had medical records, the point estimates for each type of event were somewhat strengthened relative to the results with all subjects included, and the CI largely overlapped those from our main analyses (see supplementary table S2).

**Table 3:** Relationship between working as a cane cutter and dysuria, heat events, and muscle events among male Nicaraguan sugarcane workers.

Medical outcome	Cane cutter (Person-months =1417)		Other job in sugarcane (Person-months=5840)		P-value ^b^	Crude OR ^c^ (95% CI)	Age-adjusted OR ^d^ (95% CI)
	
	Events N	Proportion months ^a^	Events N	Proportion months ^a^			
Dysuria (N=160)	64	0.045	96	0.016	<0.0001	2.47 (1.59–3.81)	2.40 (1.56–3.68)
Heat events							
Primary definition (N=21)	8	0.006	13	0.002	0.05	2.63 (1.01–6.88)	2.77 (0.99–7.77)
Primary & secondary definitions (N=44)	13	0.009	31	0.005	0.09	1.77 (0.87–3.57)	1.79 (0.83–3.85)
Muscle events							
Primary definition (N=16)	4	0.003	12	0.002	0.53	1.40 (0.47–4.21)	1.70 (0.57–5.07)
Primary & secondary definitions (N=21)	5	0.004	16	0.003	0.58	1.29 (0.43–3.84)	1.52 (0.52–4.49)

a Proportion months event occurred.

b P-value from Chi-square or Fisher’s Exact (for heat primary only definition and muscle primary only and primary and secondary definitions) test comparing proportion of medical events experienced during months worked as cane cutter compared with months worked in other sugarcane job.

c Logistic Regression using GEE method.

d Logistic Regression using GEE method, age adjusted.

In table 4, we present medical events, person-months, and proportion of months in which events were experienced by job title. Although cane cutters experienced the highest proportion of months with dysuria, we note that nonetheless dysuria occurred in all job types. There were only 35 (0.4%) person-months contributed while working as a seed cutter and only one dysuria event, preventing meaningful interpretation of the occurrence among seed cutters in our study.

**Table 4:** Medical events and crude incidence by job among 242 male Nicaraguan sugarcane workers.

Job	Person-months	Dysuria		Primary heat event		Primary muscle event	
			
	N=7257	Events N=160	Proportion months ^a^	Events N=21	Proportion months ^a^	Events N=16	Proportion months ^a^
Cane cutter	1417	64	0.045	8	0.006	4	0.003
Cane Gatherer	523	16	0.031	1	0.002	0	0
Factory Worker	1009	9	0.009	2	0.002	5	0.005
Field Machine Operator	1664	18	0.011	2	0.001	4	0.002
Irrigator	1075	16	0.015	1	0.001	2	0.002
Pesticide Applicator	894	25	0.028	2	0.002	0	0
Seed cutter	35	1	0.029	0	0	0	0
Other	640	11	0.017	5	0.008	1	0.002

a Proportion months event occurred

## Discussion

We found that the odds of dysuria were elevated for cane cutters compared with other jobs in the sugarcane industry. While observing that cane cutters experienced dysuria was not surprising, we also observed that 30% of those holding other jobs in the sugarcane industry experienced dysuria. These results support the hypothesis that cane cutters experience more events of dysuria, potential precursors to MeN, but also highlight that workers holding other jobs in sugarcane may experience dysuria. The odds of heat events were also elevated among cane cutters compared with other jobs in the sugarcane industry, though event numbers were only one-tenth of the number of dysuria events and therefore produced wide confidence intervals.

Dysuria may occur in the setting of volume depletion (15, 23) and may reflect mechanical trauma from possible crystalluria stemming from work-related heat stress (20). Dehydration and volume depletion result in very concentrated urine with supersaturated minerals in an acidic environment promoting crystal formation (14, 33). Urinary crystalline structures may damage kidney tubules and induce an inflammatory response (14, 33) as well as result in dysuria (34). It is also possible that dysuria does not play a direct role in the eventual development of MeN but rather represents a marker of heat stress, which causes kidney damage through other pathways. Critically, existing evidence outside the specific context of MeN suggests that acute injury to the kidney can affect risk of CKD over time. For example, AKI has been found to increase risk of advanced CKD 3- to 8-fold, even in the absence of preexisting CKD (35–37).

The present study provides a view of how frequently workers seek medical care for dysuria. Dysuria (or chistata) has been reported as a common symptom among sugarcane workers (5, 23, 38, 39). A cross-sectional study that relied on self-report also found that more cane cutters (25.8%) than non-cane cutters (12.7%) reported experiencing dysuria (23). Another approach to examining potential for dysuria has involved identifying crystalluria through the use of urine samples collected at defined time points. For example, urine samples collected from cane cutters near the end of the harvest season revealed crystalluria, primarily uric acid crystals (10, 14, 22), and urinary kidney injury biomarkers were elevated compared with pre-harvest measures (9).

For each of the three outcomes investigated in the present study, several factors may have influenced our ability to observe associations. Workers needed to seek medical care for the condition, and if the condition was not severe enough to seek treatment, we would have missed those events. However, our review of medical records did identify lesser symptoms reflective of our outcomes, suggesting that workers sought care for conditions of varying severity. Among those who sought care, misclassification was also possible, meaning that we classified some individuals with and without each outcome incorrectly. Dysuria is distinct in its presentation and perhaps the least likely to misclassify. For heat events, more events were based on symptoms rather than specific diagnosis of a heat condition. Similarly, muscle events indicative of rhabdomyolysis proved the most challenging to classify. Understandably, muscle injuries are common in this physical work environment. It is not clear whether the generalized muscle tenderness that is most prominent in rhabdomyolysis would be sufficiently severe to cause workers to seek medical care. For both heat and muscle events, our medical records review resulted in a large potential number of events, though many of these were excluded due to insufficient information from the visit that the event of interest occurred, or any indication that events may be caused by another factor such as viral illness, trauma, or specific muscle injury. In our classification of outcomes, we limited our probability of including false negatives. It is possible that our exclusion criteria resulted in leaving off events that were true events of interest, though loosening these criteria would also have resulted in increasing the opportunity for non-events to be counted as events. Inclusion of events meeting the secondary definition weakened results for heat and muscle events. In addition, it is feasible that the symptoms and diagnoses in the medical records are not distinct enough for characterizing these events of interest retrospectively. For each outcome, non-differential misclassification potentially biased results towards the null as we have no reason to think that clinic staff would treat the conditions differently by job title.

Our analyses included 43 workers who did not have a medical record, and we assumed they did not seek medical attention at the clinic nor did they experience any outcome of interest. Supporting that possibility is that workers without medical visits had shorter work durations and may not have needed medical attention during that more limited period of work. However, another possibility is that medical records for some workers were misplaced. We note that sensitivity analyses excluding the workers without medical visits did not change the results. Alternatively, given that cane cutters made up a higher percentage of those without a medical record, if we are missing events of interest in those without medical records, it is possible that our analyses may be underestimating the associations with cane cutters.

This study was comprised of active workers. These workers were prevalent hires, a phrase used in occupational epidemiology to describe workers who worked prior to the start of follow-up. We did not capture incident hires – those followed from the time of hire, which occurs during the study observation period. Prevalent hires represent a potential source of left truncated data, as they may not be representative of all those who worked prior to the start of follow-up. In prior research on occupational cohorts, prevalent hires were found to induce a downward bias on the observed association (40). However, much of the previous literature was focused on industries where there was more potential for longer work tenures and analyses focused on long-term exposure and chronic diseases. In the present analysis, both our exposure and outcomes were short-term – occurring within a month window (eg, whether experienced dysuria during a month worked as cane cutter or other job). Also, unlike a chronic disease, dysuria is a transient event from which there is short-term recovery. It is unclear if these conditions would lessen a potential impact of left truncation. Ideally, future studies of dysuria among workers would include incident hires, which would facilitate understanding the impact of left truncation.

There are other potential sources of bias. Because sampling was conducted on those actively working at the company, workers with a longer duration of work had more opportunities to be sampled and be represented in the study population. This strategy may result in length-biased sampling which may bias estimates if the occurrence of dysuria differed by length of time worked (41). In addition, the healthy worker survivor effect may have occurred, where less healthy workers stopped working or changed jobs. We observed that those who ever worked as a cane cutter during follow-up worked fewer months compared with those who did not work as a cane cutter during follow-up. This may have resulted in an underestimation of the association between dysuria and working as a cane cutter.

Our study, spanning up to 13.5 years of follow-up represents the longest analytical time period for a study of urinary tract events among workers at elevated risk of MeN, to our knowledge. Additionally, few longitudinal studies have examined whether sugarcane workers experience repeated symptoms of dysuria, heat stress, and muscle injury (19, 23, 38). Quantifying the occurrence of these events across time and examining their relationship with cane cutting sheds light on one of the leading hypotheses of MeN, in which recurrent subclinical kidney injury may result in kidney damage, eventually leading to clinically recognized kidney dysfunction (13, 14, 16, 17, 19). Our study is also unique in that it chronicles medical visits during the calendar years 1997–2010. This captures, in part, the experience prior to larger scale prevention efforts by sugarcane companies. It was only towards the end of this time period that some sugarcane companies in this region of Nicaragua began to make a series of changes in work practices aimed at decreasing the occurrence of heat stress among cane cutters. Future studies could compare event rates of dysuria by job type before and after the implementation of such heat stress prevention measures.

### Concluding remarks

These findings provide quantitative estimates over an extended period of time that cane cutters were more likely to experience events suspected of placing them at increased risk of developing MeN. More research involving populations at risk of MeN should examine other jobs in the sugarcane industry more closely, including potential prevention strategies (11).

### Funding

This work was supported by the Centers for Disease Control and Prevention [R21 OH 011120 (TS, DB, DW, YN, NLM, KMA)]; National Institutes of Health training grant [T32 ES014562 (RLL)]; U.S. Environmental Protection Agency [STAR Fellowship Assistance agreement no. FP-91764901-0 (RLL)]; and the Spanish Society of Epidemiology and the Instituto de Salud Carlos III [Enrique Najera predoctoral grant to (ORR)]. Its contents are solely the responsibility of the authors and do not necessarily represent the official views of the Centers for Disease Control and Prevention, the Department of Health and Human Services, or the other affiliated institutions. The EPA does not endorse any products or commercial services mentioned in this publication.

Funding for the original data collection was the result of a mediation process convened by the Compliance Advisor/Ombudsman (CAO) of the World Bank Group, between Nicaragua Sugar Estates Limited (NSEL) and Asociación de Chichigalpa por la Vida (ASOCHIVIDA). The funds were provided by the CAO and the Comité Nacional de Productores de Azúcar (CNPA), of which NSEL is a member, for the purpose of conducting a feasibility study. The funder had no influence on the study design, data collection, data analysis or interpretation, or writing the final report. The final report of the feasibility study is available at http://www.cao-ombudsman.org/cases/document-links/documents/BU_CohortPilotStudyReport_Jan2012_ENGLISH.pdf (accessed December 13, 2016).

### Conflict of interest

For the present study, lead author and data analyst (TLS), senior author (KMA), and other co-investigators (ARL, NLM, DEW, JSK, AA, YM, MPL, RLL, MW, VEM) have no conflicts of interest.

JJA, DLP, and DRB are conducting a separate research project into occupational causes of Mesoamerican nephropathy that received funding from Azucareros del Istmo Centroamericano (AICA), an industry association of sugar cane producers, through an unrestricted gift to Boston University. The agreement with AICA states that these researchers are conducting independent research and AICA cannot influence study design, data collection, data analysis or interpretation, publications, or decision to submit for publication.

## Supplementary material

Supplementary material

## References

[1] Correa-Rotter R, García-Trabanino R (2019). Mesoamerican Nephropathy. Semin Nephrol.

[2] O'Donnell JK, Tobey M, Weiner DE, Stevens LA, Johnson S, Stringham P (2011). Prevalence of and risk factors for chronic kidney disease in rural Nicaragua. Nephrol Dial Transplant.

[3] Weiner DE, McClean MD, Kaufman JS, Brooks DR (2013). The Central American epidemic of CKD. Clin J Am Soc Nephrol.

[4] Torres C, Aragón A, González M, López I, Jakobsson K, Elinder CG (2010). Decreased kidney function of unknown cause in Nicaragua:a community-based survey. Am J Kidney Dis.

[5] Wesseling C, Aragón A, González M, Weiss I, Glaser J, Rivard CJ (2016). Heat stress, hydration and uric acid:a cross-sectional study in workers of three occupations in a hotspot of Mesoamerican nephropathy in Nicaragua. BMJ Open.

[6] Raines N, González M, Wyatt C, Kurzrok M, Pool C, Lemma T (2014). Risk factors for reduced glomerular filtration rate in a Nicaraguan community affected by Mesoamerican nephropathy. MEDICC Rev.

[7] Gonzalez-Quiroz M, Smpokou ET, Silverwood RJ, Camacho A, Faber D, Garcia BR (2018). Decline in Kidney Function among Apparently Healthy Young Adults at Risk of Mesoamerican Nephropathy. J Am Soc Nephrol.

[8] Peraza S, Wesseling C, Aragon A, Leiva R, García-Trabanino RA, Torres C (2012). Decreased kidney function among agricultural workers in El Salvador. Am J Kidney Dis.

[9] Laws RL, Brooks DR, Amador JJ, Weiner DE, Kaufman JS, Ramírez-Rubio O (2016). Biomarkers of Kidney Injury Among Nicaraguan Sugarcane Workers. Am J Kidney Dis.

[10] Wesseling C, Aragón A, González M, Weiss I, Glaser J, Bobadilla NA (2016). Kidney function in sugarcane cutters in Nicaragua--A longitudinal study of workers at risk of Mesoamerican nephropathy. Environ Res.

[11] Glaser J, Hansson E, Weiss I, Wesseling C, Jakobsson K, Ekström U (2020). Preventing kidney injury among sugarcane workers:promising evidence from enhanced workplace interventions. Occup Environ Med.

[12] Hansson E, Glaser J, Weiss I, Ekström U, Apelqvist J, Hogstedt C (2019). Workload and cross-harvest kidney injury in a Nicaraguan sugarcane worker cohort. Occup Environ Med.

[13] Correa-Rotter R, Wesseling C, Johnson RJ (2014). CKD of unknown origin in Central America:the case for a Mesoamerican nephropathy. Am J Kidney Dis.

[14] Roncal-Jimenez C, García-Trabanino R, Barregard L, Lanaspa MA, Wesseling C, Harra T (2016). Heat Stress Nephropathy From Exercise-Induced Uric Acid Crystalluria:A Perspective on Mesoamerican Nephropathy. Am J Kidney Dis.

[15] Ramirez-Rubio O, Brooks DR, Amador JJ, Kaufman JS, Weiner DE, Scammell MK (2013). Chronic kidney disease in Nicaragua:a qualitative analysis of semi-structured interviews with physicians and pharmacists. BMC Public Health.

[16] Brooks DR, Ramirez-Rubio O, Amador JJ (2012). CKD in Central America:a hot issue. Am J Kidney Dis.

[17] Madero M, Sarnak MJ, Wang X, Greene T, Beck GJ, Kusek JW (2009). Uric acid and long-term outcomes in CKD. Am J Kidney Dis.

[18] Hansson E, Glaser J, Jakobsson K, Weiss I, Wesseling C, Lucas RA (2020). Pathophysiological Mechanisms by which Heat Stress Potentially Induces Kidney Inflammation and Chronic Kidney Disease in Sugarcane Workers. Nutrients.

[19] Paula Santos U, Zanetta DM, Terra-Filho M, Burdmann EA (2015). Burnt sugarcane harvesting is associated with acute renal dysfunction. Kidney Int.

[20] Stallings T, Aschengrau A, Riefkohl A, Ramirez-Rubio OR, Brooks D, Weiner D (2015). Medical Visits Among Nicaraguan Sugarcane Workers:Uncommon UTI Diagnoses and Subclinical Findings. Ann Epidemiol.

[21] McClean M, Amador JJ, Laws R, Kaufman JS, Weiner DE, Rodriguez JM (2012). Biological Sampling Report:Investigating biomarkers of kidney injury and chronic kidney disease among workers in Western Nicaragua.

[22] García-Trabanino R, Jarquín E, Wesseling C, Johnson RJ, González-Quiroz M, Weiss I (2015). Heat stress, dehydration, and kidney function in sugarcane cutters in El Salvador--A cross-shift study of workers at risk of Mesoamerican nephropathy. Environ Res.

[23] Crowe J, Nilsson M, Kjellstrom T, Wesseling C (2015). Heat-related symptoms in sugarcane harvesters. Am J Ind Med.

[24] Efstratiadis G, Voulgaridou A, Nikiforou D, Kyventidis A, Kourkouni E, Vergoulas G (2007). Rhabdomyolysis updated. Hippokratia.

[25] Torres PA, Helmstetter JA, Kaye AM, Kaye AD (2015). Rhabdomyolysis:pathogenesis, diagnosis, and treatment. Ochsner J.

[26] Vanholder R, Sever MS, Erek E, Lameire N (2000). Rhabdomyolysis. J Am Soc Nephrol.

[27] Bosch X, Poch E, Grau JM (2009). Rhabdomyolysis and acute kidney injury. N Engl J Med.

[28] Wegman DH, Apelqvist J, Bottai M, Ekstrom U, Garcia-Trabanino R, Glaser J (2018). Intervention to diminish dehydration and kidney damage among sugarcane workers. Scand J Work Environ Health.

[29] Aschengrau A, Brooks DR, McSorley E, Riefkohl A, Applebaum K, Amador JJ (2012). Cohort Pilot Study Report:Evaluation of the Potential for an Epidemiologic Study of the Association between Work Practices and Exposure and Chronic Kidney Disease at the Ingenio San Antonio (Chichigalpa, Nicaragua). Boston University School of Public Health report to the Compliance Advisor Ombudsman, World Bank.

[30] Levey AS, Stevens LA, Schmid CH, Zhang YL, Castro AF, Feldman HI (2009). CKD-EPI (Chronic Kidney Disease Epidemiology Collaboration). A new equation to estimate glomerular filtration rate. Ann Intern Med.

[31] Levey AS, Coresh J, Balk E, Kausz AT, Levin A, Steffes MW (2003). National Kidney Foundation. National Kidney Foundation practice guidelines for chronic kidney disease:evaluation classification, and stratification. Ann Intern Med.

[32] Zeger SL, Liang KY (1986). Longitudinal data analysis for discrete and continuous outcomes. Biometrics.

[33] Mulay SR, Anders HJ (2016). Crystallopathies. N Engl J Med.

[34] Kopp JB, Miller KD, Mican JA, Feuerstein IM, Vaughan E, Baker C (1997). Crystalluria and urinary tract abnormalities associated with indinavir. Ann Intern Med.

[35] Lo LJ, Go AS, Chertow GM, McCulloch CE, Fan D, Ordoñez JD (2009). Dialysis-requiring acute renal failure increases the risk of progressive chronic kidney disease. Kidney Int.

[36] Amdur RL, Chawla LS, Amodeo S, Kimmel PL, Palant CE (2009). Outcomes following diagnosis of acute renal failure in U.S. veterans:focus on acute tubular necrosis. Kidney Int.

[37] Maioli M, Toso A, Leoncini M, Gallopin M, Musilli N, Bellandi F (2012). Persistent renal damage after contrast-induced acute kidney injury:incidence, evolution, risk factors, and prognosis. Circulation.

[38] Bodin T, García-Trabanino R, Weiss I, Jarquín E, Glaser J, Jakobsson K (2016). WE Program Working Group. Intervention to reduce heat stress and improve efficiency among sugarcane workers in El Salvador:Phase 1. Occup Environ Med.

[39] Kupferman J, Amador JJ, Lynch KE, Laws RL, López-Pilarte D, Ramírez-Rubio O (2016). Characterization of Mesoamerican Nephropathy in a Kidney Failure Hotspot in Nicaragua. Am J Kidney Dis.

[40] Applebaum KM, Malloy EJ, Eisen EA (2011). Left truncation, susceptibility, and bias in occupational cohort studies. Epidemiology.

[41] Nowell C, Stanley LR (1991). Length-biased sampling in mall intercept surveys. J Mark Res.

