# Supplementary material for: Dysuria, heat stress, and muscle injury among Nicaraguan sugarcane workers at risk for Mesoamerican nephropathy
Source: Scand J Work Environ Health. 2021 Jun 29;47(5):377–86. doi: 10.5271/sjweh.3963 (PMC8259701; doi:10.5271/sjweh.3963)
Supplement: Supplementary material [file SJWEH-47-377-S001.pdf]

Dysuria, heat stress, and muscle injury among Nicaraguan sugarcane workers at risk for Mesoamerican nephropathy[1]

by Tiffany L Stallings, PhD,[2] Alejandro Riefkohl Lisci, MD, Nathan L McCray, MPH, Daniel E Weiner, MD, James S Kaufman, MD, Ann Aschengrau, ScD, Yan Ma, PhD, Michael P LaValley, PhD, Oriana Ramírez-Rubio, MD, Juan Jose Amador, MD, Damaris López-Pilarte, Rebecca L Laws, PhD, Michael Winter, MPH, V Eloesa McSorley, PhD, Daniel R Brooks, ScD, Katie M Applebaum, ScD

1. *Supplementary material*
2. *Correspondence to: Katie M. Applebaum, The George Washington University, Milken Institute School of Public Health, Department of Environmental and Occupational Health, 950 New Hampshire Ave., NW, Suite 400, Washington, DC 20052, USA. [E-mail: kapplebaum@gwu.edu]*

Table S1. Characteristics among male Nicaraguan sugarcane workers: Stratified by whether or not had a medical record.

	Medical record n=199		No medical record n=43		
	n (%)	Median (IQR)	n (%)	Median (IQR)	p-value[a]
Characteristics at baseline					
Age[b], years					
<25	60 (30.61)		10 (52.63)		0.14
25-32	70 (35.71)		5 (26.32)		
>=33	66 (33.67)		4 (21.05)		
Employment characteristics during follow-up [c]					
Number of jobs held		1 (1)		1(1)	0.13
Work months		25 (35)		11 (24)	<0.001
Ever cane cutter	62 (31.16)		31 (72.09)		<0.001
Work months as a cane cutter		0 (5)		5 (10)	<0.001

[a] p-values comparing characteristics between ever and never cane cutters: Wilcoxon rank sum test for continuous characteristics; Chi-square test for categorical characteristics

[b] Missing age for 3 workers with medical records and 24 workers who had missing medical records

[c] Characteristics during follow-up are analyzed using one observation per worker (e.g., cumulative number of work months a worker contributed during follow-up)

Table S2. Relationship between working as a cane cutter and dysuria, heat events, and muscle events among 199 male Nicaraguan sugarcane workers who had a medical record.

	Cane cutter (Person-months=1,083)		Other job in sugarcane (Person-months=5,424)				
	Events n	Proportion months [a]	Events n	Proportion months [a]	p-value [b]	Crude OR [c] (95% CI)	Age-adjusted OR [d] (95% CI)
Dysuria (n=160)	64	0.059	96	0.018	<0.001	3.07 (2.00-4.72)	2.93 (1.93-4.46)
Heat events							
Primary definition (n=21)	8	0.007	13	0.002	0.02	3.21 (1.25-8.26)	3.25 (1.21-8.72)
Primary & secondary definitions (n=44)	13	0.012	31	0.006	0.02	2.16 (1.08-4.30)	2.13 (1.02-4.43)
Muscle events							
Primary definition (n=16)	4	0.004	12	0.002	0.32	1.71 (0.58-5.06)	1.93 (0.66-5.66)
Primary & secondary definitions (n=21)	5	0.005	16	0.003	0.38	1.57 (0.53-4.62)	1.75 (0.60-5.09)

[a] Proportion months event occurred

[b] p-value from Chi-square test or Fisher's Exact (primary heat definition, primary heat only definition, and primary & secondary heat definition) test comparing proportion of medical events experienced during months worked as cane cutter compared with working in other sugarcane job

[c] Logistic Regression using GEE method

[d] Logistic Regression using GEE method, age adjusted
